# Interpretation of Controversial Teratogenic Findings of Drugs Such As Phenobarbital

**DOI:** 10.5402/2011/719675

**Published:** 2011-09-04

**Authors:** Andrew E. Czeizel, Istvan Dudás, Ferenc Bánhidy

**Affiliations:** ^1^Foundation for the Community Control of Hereditary Diseases, Budapest 1026, Hungary; ^2^Second Department of Obstetrics and Gynecology, School of Medicine, Semmelweis University, Budapest 1085, Hungary

## Abstract

*Objective*. To check the debated association between phenobarbital treatment during pregnancy and risk for congenital abnormalities (CAs) in their children. *Study Design*. It is a comparison of phenobarbital treatment in the mothers of cases with CA and matched controls without CAs in the Hungarian Case-Control Surveillance System of Congenital Abnormalities. *Results*. Of 22,843 cases with CA, 149 (0.65%) had mothers with phenobarbital treatment, while of 38,151 control newborn infants without CA, 209 (0.55%) were born to mothers with phenobarbital treatment (100–400 mg daily) (OR with 95% CI : 1.3, 1.1–1.7). Of 16 CA groups, only hypospadias had a higher risk after phenobarbital treatment in the critical period of this CA (OR with 95% CI : 2.4, 1.1–5.4). However, if only medically recorded phenobarbital treatments were evaluated and multiple testing bias was considered, this association would disappear. *Conclusions*. This study stresses the importance of the exclusion of recall bias and multiple testing bias.

## 1. Introduction

Phenobarbital has been used widely in clinical practice as a sedative and anticonvulsant since 1912 [1] among pregnant women as well. Phenobarbital crosses the placenta to the fetus; however, this transfer is influenced by the duration of treatment, pregnancy age, and arterial blood pH [2]. Previous studies showed controversial results regarding the teratogenic effect of phenobarbital [3–5], but the fetal risk of phenobarbital was classified by the FDA in the pregnancy category D (“There is positive evidence of human fetal risk, but the benefits from use in pregnant women may be acceptable despite the risk …”) [6]. Previously, we evaluated the possible teratogenic effect of phenobarbital in the offspring of pregnant women in the dataset of the Hungarian Case-Control Surveillance System of Congenital Abnormalities [7], and an association of oral phenobarbital treatment with a higher risk of structural birth defects, that is, congenital abnormalities (CAs) was not found (OR with 95% CI: 1.1, 0.7–1.7). On the other hand, we used the disaster epidemiological model for the evaluation of phenobarbital teratogenicity in the children of 88 pregnant women who attempted suicide with extremely large doses of phenobarbital [8]. 

However, CAs cannot be regarded as a single homogeneous disorder group because teratogenic factors such as drugs do not uniformly increase the rates of all CAs but rather tend to increase the occurrence of one or a limited number of specific CAs [9]. Thus, the objective of this study was to evaluate again the possible association between oral phenobarbital treatment in pregnant women and 16 CA-groups separately in their offspring in the population-based large dataset of the Hungarian Case-Control Surveillance System of Congenital Abnormalities (HCCSCA) [10] to understand better the controversial findings of previous studies.

## 2. Material and Methods

The HCCSCA is based on the comparison between the rate of exposures (i.e., phenobarbital in the study) in the mothers of cases and controls during the study pregnancy.

The first step was the selection of *cases *with different CA from the data set of the Hungarian Congenital Abnormality Registry (HCAR), 1980–1996 [11] for the HCCSCA. Reporting of CAs is mandatory for physicians from the birth until the end of first postnatal year to the HCAR. Most cases with CA are reported by obstetricians and paediatricians. In Hungary, practically all deliveries take place in inpatient obstetric clinics, and the birth attendants are obstetricians. In addition, all infants affected with CA are treated in the neonatal units of inpatient obstetric clinics, or in various general and special (surgical, cardiologic, orthopaedic, etc.) inpatient and outpatient paediatric clinics. Autopsy was mandatory for all infant deaths and common (about 80%) in stillborn fetuses during the study period. Pathologists sent a copy of the autopsy report to the HCAR if defects were identified in stillbirths and infant deaths. Since 1984, fetal defects diagnosed in prenatal diagnostic centres with or without termination of pregnancy have also been included into the HCAR. The total (birth + fetal) prevalence of cases with CA diagnosed from the second trimester of pregnancy through the age of one year was 35 per 1000 *informative offspring *(liveborn infants, stillborn fetuses, and electively terminated malformed fetuses) in the HCAR, 1980–1996 [11], and about 90% of major CAs were recorded during the 17 years of the study period [12]. 

The major objective of the HCCSCA is a postmarketing surveillance of drug teratogenicity [10]; therefore (i) cases reported after three months of birth or pregnancy termination were excluded. The longer time between birth or pregnancy termination and data collection decreases the accuracy of information about pregnancy history. However, 77% of cases were reported during the first three-month time window. (ii) Three mild CAs (such as congenital dysplasia of hip, congenital inguinal hernia, and large hemangioma), and (iii) CA syndromes caused by major gene mutations or chromosomal aberrations with preconceptional origin were also excluded. 

The second step was to ascertain appropriate *controls *from the National Birth Registry of the Central Statistical Office for the HCCSCA. Controls were defined as newborn infants without CA. In general, two controls were matched to every case according to sex, birth week in the year when the case was born, and district of parents' residence. These controls were selected on the basis of case list forwarded by the coworkers of the HCAR to the administrators of the National Birth Registry in each quarter of the year, and after the selection of controls they provided them with name and address for the coworkers of the HCAR. 

The third step was to obtain the necessary *maternal and exposure *data from three sources as follows.

**(1) Prospective Medically Recorded Data**

An explanatory letter was mailed to mothers immediately after the selection of cases and controls, and mothers were asked to send us the *prenatal maternity logbook *and other *medical records *particularly discharge summaries concerning their diseases during the study pregnancy and their child's CA. These documents were sent back after three weeks. Prenatal care was mandatory for pregnant women in Hungary (if somebody would not visit prenatal care clinic, she did not receive a maternity grant and leave), thus nearly 100% of pregnant women visited prenatal care clinics, an average of 7 times in their pregnancies. The first visit was between the 6th and 12th gestational weeks. The task of obstetricians is to record all pregnancy complications, maternal diseases, and related drug prescriptions in the prenatal maternity logbook.

**(2) Retrospective Self-Reported Maternal Information**

a structured *questionnaire *with a list of medicinal products (drugs and pregnancy supplements) and diseases plus a printed informed consent form were also mailed to the mothers. The questionnaire requested information on pregnancy complications and maternal diseases, on medicinal products taken during pregnancy according to gestational months, and on family history of CAs. To standardize the answers, mothers were asked to read the enclosed lists of medicinal products and diseases as a memory aid before they filled in the questionnaire. We also asked mothers to give a signature for informed consent form which permitted us to record their names and addresses in the HCCSCA.

The mean + S.D. time elapsed between the birth or pregnancy termination and the return of the “information package” (questionnaire, logbook, discharge summary, and informed consent form) in our prepaid envelope was 3.5 + 1.2 and 5.2 + 2.9 months in the case and control groups, respectively.

**(3) Supplementary Data Collection**

Regional nurses were asked to visit all nonrespondent case mothers. Regional nurses helped mothers to fill in the same questionnaire used in the HCCSCA; they evaluated the available medical records; in addition, they obtained data regarding smoking (cigarette/day) and drinking habit through cross-interview of mothers and fathers or other close relatives living together, and the so-called family consensus was recorded; finally, asked mothers to sign informed consent form. Regional nurses did not visit all nonrespondent control mothers because committee on ethics considered this followup to be disturbing to the parents of healthy children. Thus, only 200 nonrespondent and 600 respondent control mothers selected randomly were visited and evaluated in two validation studies [13, 14]. The difference of home visit in nonrespondent families between the groups of cases and controls explains mainly that the number of controls per cases is 1 : 1.7 instead of 1 : 2.0.

The flow of cases from the HCAR and controls from the Central Statistical Office to the HCCSCA and the achievement of final data set were published previously [15]. Overall, the necessary information was available on 96.3% of cases (84.4% from reply to the mailing, 11.9% from the nurse visit) and 83.0% of the controls (81.3% from reply, 1.7% from visit). Informed consent form was signed by 98% of mothers; names and addresses were deleted in the rest of subjects.

The fourth step of the study was the detailed evaluation of exposure studies, that is, *phenobarbital *intake from 11 approaches.

1. The source of information: phenobarbital treatments (a) only from the prenatal maternity logbooks and/or other medical records; (b) only from the questionnaire, and (c) concordant data from both medical records and the questionnaire.
2. The type of treatment: two groups were differentiated: (a) phenobarbital alone and (b) phenobarbital plus other drugs.
3. The route of administration: in Hungary, phenobarbital was available in the tablet: Sevenal (Alkaloida) containing 100 mg for oral treatment and as ampoule Sevenal (Chinoin), but parenteral treatment did not occur in the data set of the HCCSCA.
4. The dose of phenobarbital treatment: the recommended oral treatment is 1-2 tablets, that is, 100–200 mg per day.
5. The duration of treatment.
6. Maternal diseases, that is underlying medical conditions as confounders.
7. Pregnancy complications.
8. Other drug uses as confounders.
9. Pregnancy supplements. The use of pregnancy supplements may indicate the level of pregnancy care, and indirectly may show the socioeconomic status and the motivation of mothers to prepare and/or to achieve a healthy baby. In addition, it is necessary to consider folic acid and folic acid-containing multivitamins in the evaluation of preventable CAs [16–20].
10. The *gestational age *was calculated from the first day of the last menstrual period. Three time intervals were considered: (i) first month of gestation because it is before the organogenesis, the first two weeks are before conception, while the third and fourth weeks comprise the pre- and implantation period of zygotes and blastocysts including omnipotent stem cells, and it explains the “all-or-nothing effect” rule, that is, total loss or normal further development. Thus, CAs cannot be induced by environmental agents in the first month of gestation; (ii) the second and third months of gestation. This is the most sensitive, the so-called critical period for major CAs; (iii) the fourth through ninth months of gestation, that is, pregnancy after the organ-forming period.
11. Other confounding factors, such as maternal age, birth order, marital and employment status as indicator of socioeconomic status [21].

The fifth step of the protocol of the HCCSCA is the statistical analyses of data. 

*Statistical analyses *were performed using the software package SAS version 8.02 (SAS Institute Inc., Cary, NC, USA). First, the occurrence of phenobarbital treatment during the study pregnancy was compared between the two study groups (cases and controls), and crude odds ratios (OR) with 95% confidence interval (CI) were calculated. Second, frequency tables were made for the main maternal variables in order to describe the study groups of mothers with phenobarbital treatment, and of mothers without phenobarbital treatment as reference. Third, the prevalence of pregnancy complications, acute and chronic maternal diseases, other drug treatments and pregnancy supplements used during the study pregnancy were compared between case and control mothers with phenobarbital treatment, and crude OR with 95% of CI was evaluated. Fourth, the prevalence of phenobarbital treatment was evaluated according to gestational period in 16 different CA groups including at least 2 cases born to mothers with phenobarbital treatment during the second and/or third gestational months was compared with the frequency of phenobarbital treatment in their all matched control pairs, and adjusted OR with 95% CI were evaluated in a conditional logistic regression model. The latter OR were adjusted for maternal age (<25 yr versus 25–29 yr versus 30 yr or more), birth order (first delivery versus one or more previous deliveries), maternal employment status (professional managerial-skilled worker versus semiskilled worker, unskilled worker, housewife and others), and fever-related acute maternal diseases (as a dichotomous variable). The bias connected with multiple testing was limited by the use of Bonferroni method [22].

## 3. Results and Discussion

The case group consisted of 22,843 malformed newborns or fetuses (“informative offspring”) with CAs, of whom 149 (0.65%) had mothers with oral phenobarbital treatment. The total number of births in Hungary was 2,146,574 during the study period between 1980 and 1996. Thus, the 38,151 controls without CA represented (1.8%) of all Hungarian births, and among those controls, 209 (0.55%) were born to mothers treated with phenobarbital tablets (crude OR with 95% CI: 1.2, 0.9–1.5). 

The analysis of annual data showed a drastic decrease in the use of phenobarbital during the study period. 

Of 149 case mothers, 60 (40.3%), while of 209 control mothers, 88 (42.1%) had medically recorded oral phenobarbital treatments in the prenatal maternity logbooks and/or discharge summaries (*χ*_1_^2^ = 0.3; *P* = 0.59). Most pregnant women took one tablet (i.e., 100 mg) per day. Three or more tablets were used very rarely. The duration of treatment was long; the mean was 2.5 and 2.4 months in the case and control mothers, respectively, because phenobarbital was used mainly as a sedative drug in Hungary. Only one case and two control pregnant women were treated with phenobarbital alone, thus phenobarbital alone and plus other drugs were combined in our analysis. 

Most phenobarbital treatments were recorded in the 3rd gestational month both in the group of cases (no. 38; 25.7%) and controls (no. 54; 25.8%) followed by the 2nd gestational month (no. 25; 16.9% versus no. 38; 18.2%). The distribution of gestational months according to the onset of phenobarbital treatment did not show significant difference between case and control mothers (*χ*_8_^2^ = 6.7; *P* = 0.56). 

Table 1 shows the characteristics of mothers with phenobarbital treatment and without phenobarbital treatment as reference. The mean maternal age and the distribution of age groups did not show significant difference among the study groups. The mean birth order was somewhat lower in mothers with phenobarbital treatment due to larger proportion of primiparous. There was no significant difference in the proportion of marital status of mothers among the study groups. Maternal employment status showed also some differences because treated case mothers were more frequent among professional, managerial, and semiskilled workers than in untreated case mothers. These differences were not seen in control mothers. Thus, there was some but not significant difference in the distribution of employment status between case and control mothers with phenobarbital treatment (*χ*_6_^2^ : 5.3; *P* = 0.51). The proportion of smokers was not higher in phenobarbital treated case mothers (20.8% versus 21.6%) than in untreated case mothers. The proportion of regular and hard drinkers did not show significant difference among the study groups. 

Among pregnancy complications, the prevalence of threatened abortions (no. 117; 32.7% versus no. 9,896; 16.3%) and threatened preterm deliveries (no. 79; 22.1% versus no. 8,015; 13.2%) was more frequent in treated case and control mothers together because previously some medical doctors treated these pregnancy complications with phenobarbital during pregnancy in Hungary. There was no significant difference in the occurrence of other pregnancy complications between treated case and control mothers.

The incidence of acute maternal disease did not show difference between mothers with or without phenobarbital treatment either in the case or in the control group with one exception. Influenza and common cold (the latter in general with secondary complications) occurred in 47 treated case mothers (31.5%) and in 39 treated control mothers (18.7%) (OR with 95% CI: 2.0, 1.2–3.3). Among chronic maternal disorders, 95 (0.42%) case mothers and 90 (0.24%) control mothers had epilepsy, but only 4 case mothers (4.2%) and 3 control mothers (3.3%) were treated by phenobarbital (OR with 95% CI: 1.3, 0.4–8.6). Long-term phenobarbital treatment is recommended for epileptic women based on the individually defined doses. 

The evaluation of other drugs showed that drugs used for the treatment of threatened abortion (allylestrenol and promethazine) and preterm delivery (terbutaline and aminophylline) were used more frequently by phenobarbital-treated pregnant women. However, their rate did not show significant difference between case mothers and control mothers. 

Among pregnancy supplements, the use of folic acid was lower in control mothers (no. 95; 45.5% versus no. 20,680; 54.5%) and particularly in case mothers (no. 59; 39.6% versus no. 11,220; 49.4%) with phenobarbital treatment than in case and control mothers without this treatment. However, there was no difference in the occurrence of folic acid supplementation between treated case and control mothers (OR with 95% CI: 0.8, 0.5–1.2). At the comparison of other pregnancy supplements, there was no difference among the study groups. 

The objective of the study was to evaluate cases with different CAs and their *all matched controls *(Table 2). There was a somewhat higher rate of total CAs in mothers with phenobarbital treatment during the entire pregnancy compared to control mothers with this treatment (adjusted OR with 95% CI: 1.3, 1.1–1.7). However, of 16 CA groups evaluated, only hypospadias based on 31 cases showed a higher rate of phenobarbital treatment in their mothers. In the next step, phenobarbital treatment was evaluated only in the 2nd and/or 3rd gestational months, that is, the critical period of most major CAs. There was no higher risk either for the total group of CAs or for any CA group in cases born to mothers with phenobarbital treatment during this time window of the study pregnancy. However, the critical period of hypospadias is in the 3rd and/or 4th gestational months; thus, phenobarbital treatment was evaluated in this CA during this time window. This approach showed a significant association between hypospadias and phenobarbital treatment (OR with 95% CI: 2.4, 1.1–5.4).

We evaluated only medically recorded phenobarbital treatment during the third and fourth gestational months in the mothers of cases with hypospadias, and a borderline association was found between phenobarbital use and hypospadias (adjusted OR with 95% CI: 2.4, 1.0–5.4).

Finally, the Bonferroni adjustment showed that association of phenobarbital treatment in the third and/or fourth gestational months with the risk of hypospadias disappeared (*P* = 0.09). 

Of 4 cases born to epileptic mothers with phenobarbital treatment, one had undescended testis (after monotherapy), one cleft palate (after polytherapy of phenobarbital, carbamazepine, and primidone), ventricular septal defect (after polytherapy of phenobarbital, carbamazepine, and sulthiame), and multiple CA: cleft palate, microtia, ventricular septal defect, pseudohermaphroditism (after polytherapy of phenobarbital, primidone, and phenacemide). Thus, hypospadias did not occur among these cases.

## 4. Discussion

The objective of our study was to evaluate the possible association between oral phenobarbital treatment and the risk for different CAs. Our data showed only an association between hypospadias and phenobarbital treatment during the critical period of this CA group, but finally this association was not confirmed at the evaluation of only medically recorded exposure, that is, phenobarbital treatment and after the consideration of multiple testing bias. 

The teratogenic effect of anticonvulsant drugs was confirmed by several studies [4, 5]; however, different anticonvulsant drugs have different risks and spectrum of CAs [23]. Nevertheless, it had become an accepted view that epileptic pregnant women taking phenobarbital had a 2-3 times greater risk for delivering a child with CA over the general newborn population [4, 5]. In general more than one anticonvulsant drugs (the so-called polytherapy) were used in epileptic women with seizure during pregnancy; thus, it was necessary to differentiate the effect of phenobarbital from the teratogenic effect of other anticonvulsant drugs [24]. A higher prevalence of hypoplasia of nails and phalanges was found in the children of epileptic pregnant women with phenobarbital monotherapy [25]. In the MADRE surveillance project, 65 epileptic pregnant women had phenobarbital monotherapy and a higher rate of cardiovascular CAs, and orofacial cleft was detected in their children [26].

However, several studies did not confirm the teratogenic potential of phenobarbital. Fedrick [27] studied 41 epileptic women treated with phenobarbital monotherapy during the first trimester of pregnancy and did not detect a higher rate of CA in their children. Bethenod and Frederich [28] also evaluated epileptic pregnant women, and among the children of 6 pregnant women exposed to phenobarbital, only one child showed dysmorphic face. The frequency of CAs and minor anomalies was not greater than expected among the children of 1,415 women treated with phenobarbital during the first four lunar months in the study of Heinonen et al. [3]. Robert et al. [29] identified 40 epileptic pregnant women with phenobarbital treatment alone, and among their children, one was affected with ventricular septal defect while another with hypospadias. In a prospective Italian study, of 83 pregnant women with phenobarbital therapy, 4 delivered children with CAs (Fallot tetralogy in heart, hydronephrosis, inguinal hernia with umbilical hernia and congenial dislocation of the hip) [30]. Rosa et al. [31] evaluated 334 fetuses that had been exposed to phenobarbital during the first 15 trimester of pregnancy. A total of 20 (6.0%) major CAs were observed (14 expected). Six CA groups were analyzed separately on the basis of comparison of observed and expected numbers, and only cardiovascular CAs showed a higher observed number (8/3). 

Thus, the previous data did not result in unequivocal findings regarding the teratogenic effect of phenobarbital monotherapy during pregnancy. However, it is important to stress that these studies were preformed in epileptic pregnant women. Milkovich and Van Den Berg [32] studied 325 nonepileptic pregnant women with barbiturate treatments and did not find a higher rate of CAs in their children. Shapiro et al. [33] evaluated 8,000 nonepileptic pregnant women who were treated with phenobarbital during pregnancy and they did not find a higher rate of CAs in the children of mothers when the drug was taken for indications other than epilepsy. 

Our study showed a possible association between hypospadias and phenobarbital treatment in nonepileptic pregnant women. However, hypospadias show a wide spectrum of CA from coronal (minor anomaly) through glandular (mild CA) to penile, penoscrotal and scrotal (severe CA) types [34]. Our effort was to exclude minor anomalies such as coronal hypospadias from the data set of the HCCSCA, but we were not able to check all reports of cases with unspecified hypospadias. On the other hand, it is necessary to differentiate isolated and multiple-syndromic categories of hypospadias. Only cases with isolated hypospadias were evaluated in our study. The etiology of isolated hypospadias is explained by the multifactorial origin, that is, polygenic-environmental interaction [35, 36]. As far as we know, only the study of Roberts et al. [29] found a child with hypospadias after phenobarbital treatment in epileptic pregnant women. Thus, it is worth considering phenobarbital among the triggering factors of hypospadias-related polygenic system, but if we considered only medically recorded phenobarbital treatment and multiple testing bias, this association would disappear. 

Our data support therefore the statement that use of phenobarbital in nonepileptic patients does not seem to pose a significant risk for CAs [5]. Nevertheless, the controversial findings regarding the teratogenic effect of drugs used in the small clinical doses by pregnant women and modified by several confounding factors are typical in medical teratology. Another model is necessary to achieve an unequivocal conclusion. The so-called disaster epidemiological model seems to be useful to diminish this dilemma [37]. The Hungarian self-poisoning project in pregnant women, 1960–1993, included 1,044 pregnant women, and 88 surviving pregnant women used phenobarbital for their suicide attempt [8]. Doses ranged between 400 and 3,000 mg of phenobarbital. Of 88 exposed children, 12 (13.6%) were affected with CA. Of their 78 sib controls, 8 (10.3) had CAs (OR with 95% CI: 1.4, 0.3–3.5). Of 88 pregnant women, 34 attempted suicide with very large doses of phenobarbital between the 3rd and 12th postconceptional weeks, that is, during the critical period of most CAs. Three children were affected with diaphragmatic CA, multiple CA (but there was no hypospadias among component CAs) and undescended testis. Of the other 9 cases with CA, only one had penile hypospadias with strabismus, his mother attempted suicide with 1000 mg of phenobarbital and 12,000 mg of meprobamate on the 22nd gestational week of pregnancy. Thus, there was no overlapping between the critical period of hypospadias and phenobarbital abuse; therefore, the disaster epidemiological model did not confirm the possible association of phenobarbital and the higher risk of hypospadias, and in general the teratogenic effect of phenobarbital. 

Thus, it is worth summarizing that the weaknesses of previous studies resulted in controversial findings regarding the teratogenic potential of phenobarbital. First, the underlying maternal diseases, such as epilepsy, were not considered at the evaluation of phenobarbital. Though the study of Holmes et al. [38] concluded that the distinctive pattern of CAs and minor anomalies observed in infants exposed to anticonvulsants during pregnancy was caused by the drugs, rather than by epilepsy itself, we have to consider maternal diseases among confounders. 

Second, the exposure, that is, phenobarbital treatment was based on retrospective maternal information in most previous studies; therefore, recall bias might modify their results. The birth of an infant with CA is a serious traumatic event for most mothers who therefore try to find a causal explanation such as diseases or drug uses during pregnancy for CA of their babies. This does not occur after the birth of a healthy newborn infant. Thus, recall bias might inflate an increased risk for CAs. Our previous analysis showed that a case-control surveillance of this type may cause spurious association between drugs and CAs with biased OR up to a factor of 1.9 [39]. Thus, at the planning of study design it is necessary to limit recall bias by the evaluation of critical period of CAs because we expect an underreporting of phenobarbital treatment in both the critical and noncritical periods of CAs in the control group. In addition we can exclude recall bias with the use of only prospectively and medically recorded data as a gold standard. 

Third multiple comparisons may produce a noncausal association because a significant difference is expected in every 20th estimation as a result of chance. 

The strengths of HCCSCA can be explained by the population-based and large data set of 358 pregnant women with phenobarbital treatment in ethnically homogeneous European (Caucasian) Hungarian population. In addition potential confounding factors were available for analysis and additional strengths include the matching of cases to controls without CAs. The diagnosis of CAs has a good validity because cases with CA were reported by medical doctors and these diagnoses were checked by experts in the HCAR [11] and later modified, if necessary, due to the results of recent medical examinations which were available in the HCCSCA [10]. Finally, possible biases such as recall and multiple testing biases were considered. 

Of course, limitations of the data set need to be mentioned as well. (i) The response rate of case and control mothers was similar, but all nonrespondent case mothers were visited at home to collect the necessary data, while it was performed only in 200 nonrespondent control women. However, there was no difference in the distribution and occurrence of drug treatments and diseases between respondent and nonrespondent mothers in our validation study [13]. (ii) In addition, there was a longer time between the end of pregnancy and the return of information package in the control group than in the case group. However, this time difference cannot modify the medically recorded data. (iii) The HCCSCA is not appropriate to evaluate other pregnancy outcomes such as miscarriages, in addition the smoking and drinking habits were evaluated only in the subsamples of mothers visited at home on the basis of the so-called “family consensus” through personal cross interview of mothers and other members of family living together. This approach was necessary because our previous validation study indicated the unreliability of retrospective maternal information regarding their lifestyle during the study pregnancy [40]. 

In conclusion, our study showed that phenobarbital treatment did not associate with a higher risk for total CAs. Among different CA groups, there was a higher risk of cases with hypospadias born to nonepileptic pregnant women with phenobarbital treatment. However, this finding would disappear if we evaluated only medically recorded phenobarbital treatments and considered the multiple testing bias. Thus, the FDA classification of phenobarbital as the pregnancy category D is not correct.

## Figures and Tables

**Table 1:** Characteristics of mothers.

Maternal variables	Case mothers				Control mothers			
	without		with		without		with	
	phenobarbital treatment				phenobarbital treatment			
	(*N* = 22,694)		(*N* = 149)		(*N* = 37,942)		(*N* = 209)	
	no.	%	no.	%	no.	%	no.	%
Quantitative								

Maternal age (yr)								
24 or less	10,876	47.9	69	46.3	17,882	47.1	112	53.6
25–29	7,106	31.3	48	32.2	12,823	33.8	62	29.7
30 or more	4,712	20.8	32	21.5	7,237	19.1	35	16.7
Mean ± S.D.	25.5 ± 5.3		25.7 ± 5.5		25.5 ± 4.9		25.0 ± 4.8	
Birth order								
1	10,636	46.9	72	48.3	18,095	47.7	114	54.6
2 or more	12,058	53.1	77	51.7	19,847	52.3	95	45.4
Mean ± S.D.	1.9 ± 1.1		1.7 ± 0.9		1.7 ± 0.9		1.6 ± 0.9	

Categorical								

Unmarried	1,264	5.6	5	3.4	1,462	3.9	9	4.3
Employment status								
Professional	1,882	8.3	19	12.7	4,335	11.4	18	8.6
Managerial	4,931	21.7	37	24.8	10,076	26.6	58	27.7
Skilled worker	6,291	27.7	38	25.5	11,624	30.6	66	31.6
Semiskilled worker	3,835	16.9	34	22.8	5,744	15.1	39	18.7
Unskilled worker	1,492	6.6	11	7.4	1,843	4.9	16	7.7
Housewife	2,122	9.4	6	4.0	2,034	5.4	4	1.9
Others	2,141	9.4	4	2.7	2,286	6.0	8	3.8

**Table 2:** Results of multivariate matched analysis for each case with different CAs and its all (1–3) matched controls using conditional logistic regression model to estimate the adjusted odds ratio (OR) with 95% confidence interval (CI) of oral phenobarbital treatment during the entire pregnancy and the second and/or third gestational months.

Study groups	Grand total	Second and third months				Entire pregnancy			
	no.	no.	%	Adjusted OR*	95% CI	no.	%	Adjusted OR*	95% CI
Isolated CAs									
Neural-tube defects	1,202	4	0.3	3.9	0.6–23.4	11	0.9	2.3	0.8–6.1
Cleft lip ± palate	1,374	6	0.4	2.3	0.7–7.9	12	0.9	1.8	0.8–4.3
Posterior cleft palate	582	2	0.3	—	—	3	0.5	1.2	0.3–5.5
Intestinal atresia/stenosis	153	1	0.7	—	—	2	1.3	3.9	0.3–46.1
Obstructive urinary CAs	271	0	0.0	—	—	2	0.7	—	—
Hypospadias	3,038	13	0.4	2.3	0.9–5.4	31	1.0	**2.6**	**1.4**–**4.8**
Undescended testis	2,051	6	0.3	1.0	0.3–2.7	16	0.8	1.7	0.8–3.4
Congenital hydrocephaly	314	1	0.3	1.4	0.1–24.9	2	0.6	0.8	0.1–5.2
Ear CAs	354	0	0.0	—	—	3	0.9	4.5	0.4–46.2
Cardiovascular CAs	4,479	4	0.1	0.3	0.1–0.9	16	0.4	0.7	0.4–1.3
Clubfoot	2,424	4	0.2	1.9	0.5–7.9	10	0.4	1.0	0.4–2.3
Limb deficiencies	548	3	0.6	0.8	0.2–3.3	4	0.7	0.9	0.2–3.3
Poly/syndactyly	1,744	8	0.5	2.6	0.9–7.6	12	0.7	1.5	0.7–3.4
Diaphragmatic CAs	243	0	0.0	—	—	4	1.7	2.3	0.5–9.6
Other isolated CAs	2,717	6	0.2	0.9	0.3–2.4	9	0.3	0.5	0.2–1.1
Multiple CAs	1,349	5	0.4	2.5	0.6–9.8	12	0.9	1.6	0.7–3.5

Total case group	22,843	63	0.3	1.3	0.9–1.9	149	0.7	**1.3**	**1.1**–**1.7**

Total control group	38,151	92	0.2	Reference		209	0.6	Reference	

*Matched OR adjusted for maternal age, birth order, maternal employment status, and high fever-related maternal diseases.

Bold numbers show significant associations.
